# Treatment of Aneurism by Compression

**Published:** 1854-04

**Authors:** 


*Treatment of Aneurism by Compression.*—In the "Medical Times and Gazette" of October 29th, and November 5th and 12th, a very interesting series of cases of external aneurism is given, which have been recently subjected to surgical treatment in the metropolitan and some of the provincial hospitals. These cases amount to twenty-five in number, and are nearly all well and accurately reported. The details of the cases furnish much information, which is very desirable for a proper estimation of the comparative value of the modes of treatment by ligature and by compression, as well as in determining the cases in which the one or the other mode of treatment is preferable.

The list comprises 23 cases of aneurism of spontaneous and 2 of traumatic origin. The seats of the aneurism of spontaneous origin were as follows:—Popliteal artery, 19; femoral, 3; radial, 1. The traumatic aneurisms were situated in the femoral and in the anterior tibial arteries. In one case a popliteal aneurism existed in each limb; and in one, double femoral aneurism existed in one artery. One case only (popliteal) occurred in the female sex.

Of these 25 cases, 23 were submitted to treatment by compression, which succeeded in curing the disease in 14 and failed in 9. Ligature of the artery without previous compression, was followed only in one case, and without success. In the cases in which compression proved successful, the artery was compressed during periods varying from three days to upwards of five months. The duration of the compression till the tumor was solidified in the different cases was as follows:—3 days, one case; 4 days, one; 8 days, three; 11 days, one; 15 days, one; 21 days, one; a month, one; 6½ weeks, one; 10 weeks, two; 15 weeks, one; and 23 weeks, one. In the case of double popliteal aneurism the cures were effected respectively after 4 and 8 days' compression.

The treatment by compression was abandoned in six cases of aneurism of the popliteal, in two of the femoral, and in one of the radial artery. The causes which led to the abandonment of the compression were nearly the same in all these cases, pain and constitutional disturbance, with œdematous or erysipelatous swelling of the limb, and increasing size of the tumor. The result of the ligature in these nine cases was that six were cured, one (a case attended by unusual peculiarities) remained *in statu quo*, and two died, one from subsequent suppuration of the sac and of the knee-joint, the other from gangrene, from the femoral vein having been wounded in the operation.

In one of the cases of traumatic aneurism (that of the anterior tibial artery) compression was successful in curing the disease; in the other, (that of the femoral artery, alluded to as a case of a peculiar kind) neither compression nor the ligature effected any change in the condition of the tumor.

This short abstract proves, in the first place, the very general confidence which now appears to be placed in the treatment by compression, or at least the general anxiety on the part of surgeons to test its merits. Very great attention and perseverance appear to have been bestowed on the treatment in nearly every case. The apparatus employed in Dublin (Carte's) was that almost invariably applied, alternated in some cases with the employment of manual pressure, or of a weight placed on the artery. The pressure was variously borne, some of the patients complaining little of its irksomeness, others passing sleepless days and nights and insisting on occasional intervals of relief from all pressure. The treatment by compression was not attended by any fatal result, nor, so far as we can perceive, by any decidedly mischievous consequence. The number of cases in which it was found necessary to abandon compression and to resort to the use of ligature is considerable (about 40 per cent.); but the proportion of cases in which the cure was effected after a short period of compression is greater, we think, than in any series of cases hitherto recorded,—and it is worthy of remark that the tumor was of comparatively small size in all the cases in which a few days of compression sufficed for the cure.

Death followed the application of the ligature in two cases of the nine in which the vessel was tied. In one of these suppuration of the sac and of the knee-joint led to the fatal result. The case does not appear to tell against the use of the ligature; for the operation was performed after the artery had been subjected to pressure for a fortnight, during which time the tumor had increased in size, the thigh had become œdematous and the patient had suffered much pain. The advocates of the use of the ligature might tell us, and with some justice, that the ligature of the artery in this case at first would probably have saved the patient, and that the result was due to the unfavorable state of the limb produced by the compression, and to the progress which the tumor had been allowed to make during the period of its employment. The second fatal result was attributable to the vein being wounded in the operation, and we think that the possible occurrence of this accident (which has many times happened) forms to a certain extent an element in the question of preference of the one or other method.

The perusal of these cases certainly tends to increase our confidence in the treatment by compression, as a means of cure which should, in the majority of cases, supersede the use of the ligature in the treatment of popliteal and femoral aneurism. Their history, too, confirms our opinion as to the class of cases in which compression is especially applicable, viz., those where the tumor is small, where it contains a considerable quantity of solid matter, and when it is not making rapid increase in size. It is true that compression has effected a cure in many cases in which these favorable conditions were not present; but we think that the general result of such cases does not present sufficiently favorable evidence to establish the superiority of the treatment by compression over that by ligature, except in the class of cases we have mentioned.—*Edinburgh Medical and Surgical Journal.*

